# A Qualitative Exploration of Sport and Social Pressures on Elite Athletes in Relation to Disordered Eating

**DOI:** 10.3389/fpsyg.2021.633490

**Published:** 2021-04-23

**Authors:** Hannah Stoyel, Russell Delderfield, Vaithehy Shanmuganathan-Felton, Alex Stoyel, Lucy Serpell

**Affiliations:** ^1^University College London, London, United Kingdom; ^2^University of Bradford, Bradford, United Kingdom; ^3^University of Roehampton London, London, United Kingdom; ^4^University of Portsmouth, Portsmouth, United Kingdom

**Keywords:** sport, athlete, thematic analysis, eating disorder, body image, social media

## Abstract

**Introduction:** Athletes are at increased risk of disordered eating compared to non-athletes. Inspired by previous investigation into quantitative work on an etiological model of disordered eating in athletes, the current study aimed to explore a problematic aspect of the model: athletes' lived experiences of social and sport pressures in relation to the onset of disordered eating and differing eating behaviors.

**Methods:** Nine (*N* = 9) male and female athletes representing a range of endurance sports took part in semi-structured interviews. Thematic analysis was utilized.

**Analysis:** Analysis revealed two main themes each with two corresponding subthemes (1) Conflating physical appearance and sporting ability with the subthemes of (1a) social comparison in a sporting world and (1b) societal notions of “the athlete body” and (2) Living as an athlete with the corresponding subthemes of (2a) discipline and sacrifice and (2b) the balancing act.

**Discussion:** It is the complex interaction between societal expectations as lived out in social messages and comparisons, and sport pressures that contributes to the development of disordered eating behaviors. These findings suggest that prevention and treatment of disordered eating in athletes can be applied from those already established in non-sporting realm.

## Introduction

Being part of organized sport and exercise has benefits in that it provides a sense of purpose, can build self-esteem, release endorphins, and improve health and mental outcomes (Hausenblas and McNally, 2004). However, there is consensus that athletes are at higher risk of developing subclinical and clinical eating disorders and disordered eating than the general population, especially for female athletes and for those athletes in sports that have weight class components or rely on a lean physique to achieve (Sundgot-Borgen and Torstveit, 2004; Petrie and Greenleaf, 2007; Joy et al., 2016). Athletes not only experience sport-related pressures but are also subject to broader societal pressures around weight, body image, and eating faced by the general population. These societal pressures can either compound sport pressures or oppose them, creating a climate ripe for the development of disordered eating (Sundgot-Borgen and Torstveit, 2004; Petrie and Greenleaf, 2007; Petrie et al., 2009b; Pope et al., 2015; Cooper and Winter, 2017). Similarly, sport literature has underscored a number of pressures that are specific to each sport that are often exacerbated by societal burdens as reasons why athletes suffer from clinical and subclinical eating disorders (Rudd and Carter, 2006; Petrie et al., 2009a; Anderson et al., 2012; Cooper and Winter, 2017).

**Figure 1:**
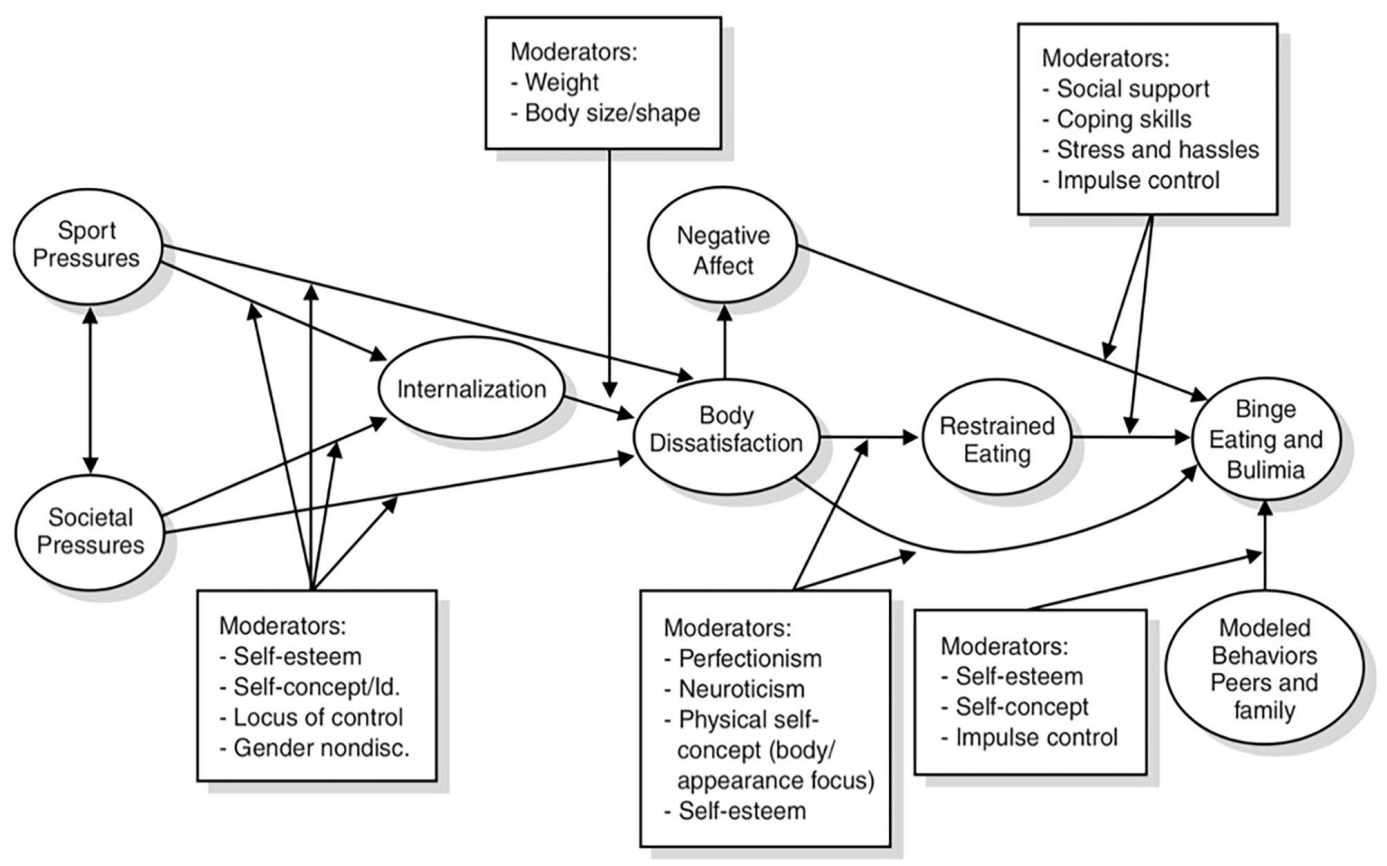
Theoretical model of disordered eating development in athletes proposed by Petrie and Greenleaf (2007).

Specifically, Petrie and Greenleaf (2007) proposed an etiological model which proposed societal and sport pressures as predictors of disordered eating (see Figure 1). This theoretical model aims to predict how sport pressure and societal pressure jointly influence the development of disordered eating behavior in the form of dietary restraint and bulimic symptomology in athletes. The model proposes that these two predictors are mediated by internalization, negative affect, modeled behaviors, and body dissatisfaction leading to restrained eating and binge eating and bulimia (see Figure 1). The two predictors in the theoretical model, sport pressure, and societal pressure are the focus of the current study.

In terms of sport pressure, research reveals several specific aspects of sport that are associated with increased rates of disordered eating, including whether the sport emphasizes a lean physique for success (lean sports) in addition to the level of competition (Petrie and Greenleaf, 2007). Lean sports, by nature, create the pressure to be thin to maximize both performance and appearance for judges and others (Smolak et al., 2000; Patel et al., 2003; Krentz and Warschburger, 2011; Chapman and Woodman, 2016).

Competitive level also has been demonstrated to be related to disordered eating risk, with research revealing that higher levels of competition increase the need for a competitive advantage, which may create a pressure that can force athletes to resort to disordered eating behaviors in an attempt to increase performance or as a coping mechanism (Swoap and Murphy, 1995; Kirk et al., 2001). Overall, weight loss has been seen by athletes to be a performance enhancer for competitive moments, and with this message being pushed by society in general, the pressure to limit body fat comes from wanting to perform well and fulfill societal expectations (Rosen et al., 1986; Sundgot-Borgen, 1994). Other sport pressures linked to disordered eating may include overt or covert comments from coaches or teammates on how appearance and physique may impact performance (Kerr et al., 2006; Petrie et al., 2009a; Scoffier et al., 2010; Kong and Harris, 2015). Upcoming competition may also play a role in creating additional sport pressure (Neves et al., 2017). Figure-hugging sport uniforms required for aerodynamics and therefore sport performance may also create pressures on athletes as they can intensify feelings of body shame and therefore encourage disordered eating behaviors (Sundgot-Borgen and Torstveit, 2004; Tylka and Hill, 2004; Cooper and Winter, 2017). These sport pressures are experienced uniquely by each athlete in their own sporting context, against a background of broader societal pressures.

Before proceeding, it is important to define what is meant by “society” in this context. For this investigation, society is constitutive of the non-athlete population, including those who are aware of competitive sport through being personally connected to it via friends, family, or fandom. There is societal pressure, independent of sport, on each gender. Broadly in Western culture, for women, societal pressure is to be thin, while for men, it is to fit a muscular ideal (Reel et al., 2013). These societal ideals presented in the environment and media have been linked to the development of eating disorders (Keery et al., 2004; Hutchinson and Rapee, 2007). Yet every sport also demands a specific body type ideal for success (Voelker et al., 2014). The desirable body type for success in many sports might contradict societal ideals. General pressures from Western media interact with body-type ideals or body-type stereotypes, which are also socially-transmitted but are specific to each sport; for example, the abdominal muscles are highly valued in swimming, whilst a lean physique is valued in long distance runners. These interactions can create a heightened risk of body dissatisfaction and disordered eating (Thompson et al., 1999; Bissell, 2004; Milligan and Pritchard, 2006; Cooper and Winter, 2017). This body dissatisfaction has been found to be closely linked to the development of disordered eating in both the general population and amongst athletes (Menzel et al., 2010; Ferreira et al., 2013; Kong and Harris, 2015). As will be seen below, an unexpected element of our findings, representing changes over the last decade of twenty-first century, is the emergent emphasis on these athletes interaction with, and influence by, social media. This was not present in the original model. Social media involves viewing photographs and related comments from online users of sharing platforms. This has been linked to problematic bodily self-judgement and to factors related to disordered eating and eating disorder symptomatology (Hobza et al., 2007; Santarossa and Woodruff, 2017).

**Figure 2:**
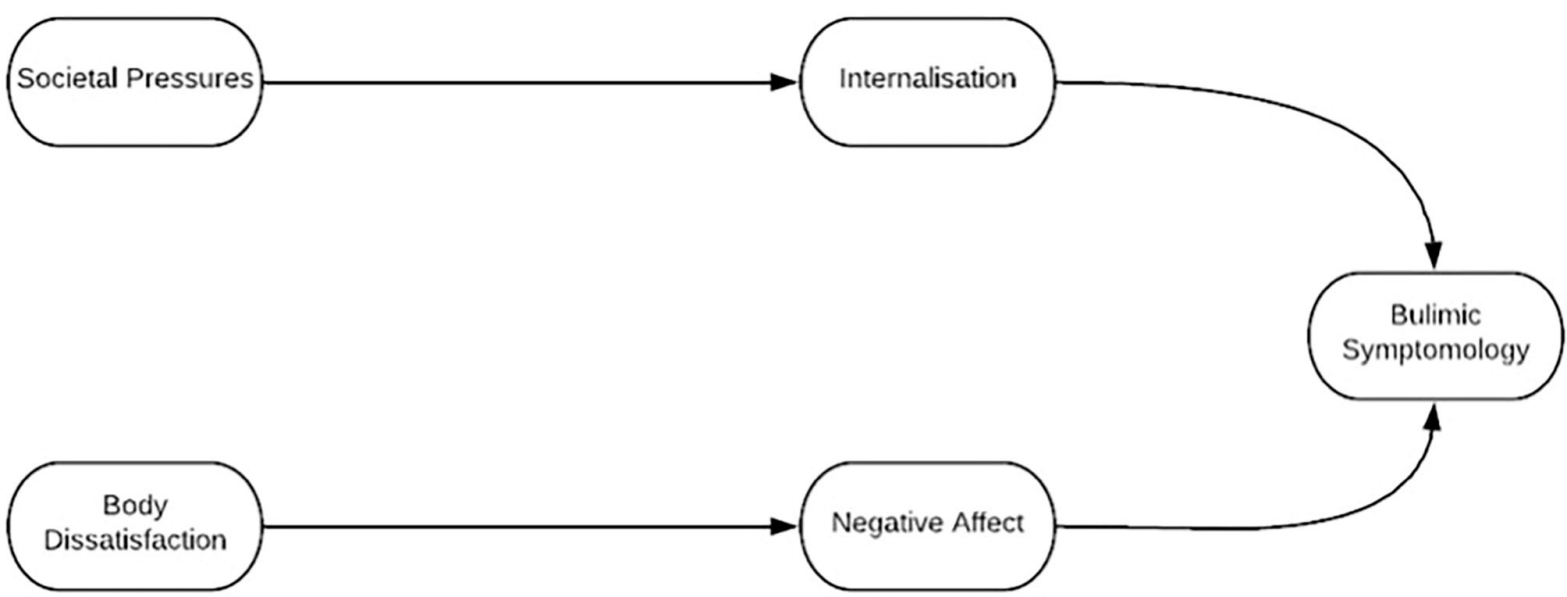
T1 model: Revised model by Stoyel et al. (2020). This model has been corrected from the first published version which coined the social pressures rather than societal pressures in the top, right bubble.

This qualitative study is part of a larger longitudinal quantitative multi-method project that investigates the risk factors of disordered eating in athletes using both quantitative and qualitative approaches. The project began by examining Petrie and Greenleaf's (2007) model with an attempt to apply this model to a sample of male and female athletes across a wide range of sports and competitive levels via quantitative research. The quantitative work studied the fit of Petrie and Greenleaf's (2007) model in more than 1,000 adult athletes using structural equation modeling. The results of this indicated that a revised model better explained disordered eating development in athletes (Stoyel et al., 2020) (see Figure 2). The quantitative study found that the predictor of sport pressures was not a significant risk factor for eating disorders in the new, applied model, but that the role of social media is a significant social pressure (Stoyel et al., 2020). The diminishing of sport pressures as a predictor of disordered eating symptoms was unanticipated and opposes anecdotal experience from sport psychologists. Increasing amounts of quantitative research have begun to support this questioning of the power of sport pressures (Shanmugam et al., 2011; de Sousa Fortes et al., 2015). What is not apparent is why this has happened. Given this lack of insight, the purpose of this qualitative study was to offer experiential insight into the role of societal (we will later argue, social) and sport pressures on athletes in relation to the development of disordered eating and related behaviors.

## Methods

### Participants

We refer to the athletes as a participant group, rather than the traditional term “sample,” as this is exploratory work and we wanted to talk to anyone who would be willing to share their experiences. Nine athletes volunteered from the main quantitative work and had previously given informed consent for all aspects of the research project. Potential participants were emailed to ask for their participation in the interview part of the project. As such, we relied on a convenience group of participants who felt comfortable discussing this poorly-understood area. The exploratory nature of the study embraces the concept of convenience in finding a participant group from the athletic community. Therefore, nine people expressed an interest and these nine were subsequently interviewed and included in this analysis. Interviews were conducted from March 2019 to June 2020. Names used henceforth are pseudonyms for these participants (see Table 1).

**Table 1:** Participant information.

**Participant**	**Gender**	**Sport**	**Age**	**EDE-Q**
Alicia	F	Orienteering	28	1.18
Ben	M	Swimming	19	0.49
Callum	M	Triathlon	44	0.85
Dan	M	Ultra-Running	30	0.49
Emily	F	Lacrosse/Rowing	19	3.42
Francesca	F	Swimming	19	1.00
Georgia	F	Mid-Distance Running	25	3.99
Henry	M	Running	33	1.73
Isabelle	F	Lacrosse/Track/Triathlon	25	1.61

All nine participants were of an elite level, competing at a national or international standard. Four of the participants were male. All participants completed the Eating Disorder Examination Questionnaire (EDE-Q), a tool that is commonly used in research and clinical work as part of a diagnostic procedure for eating disorders (Fairburn and Beglin, 2008). No participant scored above the clinical threshold of 4.0 on the EDE-Q (Lavender et al., 2010) (see Table 1). Several participants did self-disclose issues with food. Georgia and Isabelle alluded to histories of disordered eating. Alicia and Emily each reported a history of a formally diagnosed eating disorder, and Henry described a current self-diagnosed eating disorder.

### Design

This study was of qualitative design and employed deductive thematic analysis. This method allows for existing knowledge of theories to provide the backdrop for the questions and analysis of interviewees' responses (Braun and Clarke, 2012; Braun et al., 2016). Thematic analysis uses a relatively homogenous sample from which a subjective interpretation of the human experience is uncovered (Guest et al., 2012; Smith and Osborn, 2015). In this case, our group is homogenous inasmuch as they are all competing athletes The underlying ontology and epistemology were such that each participant's truth was a construction of their independent reality, and that the role of the research was to discover those experiences of independent realities unique to each participant and elaborate common themes that explored existing theory (Sparkes and Smith, 2013). The semi-structured flow and open-ended questions reflected the underlying epistemology of the inquiry and lead author (Mayan, 2016). The interviews, each of which lasted ~1 h, focused on questions related to food, bodily and performance pressures. Questions included: “Is there anything unique to being an athlete that you think makes you view food differently than a non-athlete?” and “In what ways do you feel that you live up to societal expectations of you as an athlete?” Of note, in the interviews conducted in mid-2020 (interviews with Georgia, Henry, and Isabelle), an open-ended question was added to ask about experience of Covid19 as it related to food intake, training regime, and view of food. The first author is a registered Sport Psychologist with the Health Care Professions Council, and skills from that professional experience were utilized to build rapport as well to guide as expert questioning and probing.

Eight of the nine participants were interviewed using virtual means (Skype or Zoom) (Janghorban et al., 2014). As the lead researcher of this project is both familiar with the theory surrounding eating disorders in athletes, self-reflection through journaling and supervision was employed to expose and question the researcher's preexisting assumptions and to increase the authenticity and credibility of the research (Morrow, 2005; Tracy, 2010).

After verbatim transcription, all transcripts were shared with the interviewees to ensure accuracy (Lincoln and Guba, 2000; Shenton, 2004; Tracy, 2010). Data analysis was aided using NVivo 12. Thematic analysis can be inductive or deductive, with the current study employing a deductive approach. To enhance rigor, published guidelines were utilized throughout analysis (Braun and Clarke, 2012; Braun et al., 2016). To attain complete familiarization with the data, multiple readings of the transcript were performed. The subsequent step involved coding the repeating and prominent ideas appearing in the transcripts. These initial codes were then sorted into themes and subthemes that mapped the underlying theory. When designing and reflecting upon the themes, the mutually exclusive and collectively exhaustive (MECE) principle was employed (Chillarege et al., 1992; Lee and Chen, 2018). Before discussion of the themes among the research group, additional verification of the themes took place, in which a second researcher (AS) coded the data with no influence from other researchers to ensure alternative explanations or interpretations were not missing (Smith, 2003). The first and second researcher together then reviewed, defined and named the themes. This step included data reduction, where themes perceived to be weaker or with a paucity of evidentiary extracts were eliminated or collapsed into more robust themes, as is common in qualitative analysis. This process also refines reporting so that findings are not unwieldy or inaccessible. Part of this process involves all researchers utilized a reflexive process to enhance the trustworthiness of the analysis throughout analytical engagement (Morrow, 2005; Tracy, 2010). This involved reading the team's interpretations, meeting to discuss points of convergence and divergence, debate for and against codes as they were built into themes, and refining and retaining those which were analytically defensible to the group (Nicolson, 2003; Paulus et al., 2010).

## Analysis

The qualitative study reveals two overarching themes*: conflating physical appearance and sporting ability* and *living as an athlete* (Table 2). The first theme was found to have two corresponding subthemes: the first, is *social comparison* in terms of body type between teammates, competitors, and idols within the sporting context, and the second subtheme is how the *societal notion of “the athlete body”* puts pressure on the athletes to look a certain way. The second theme, *living as an athlete*, also has two subthemes, which are the lifestyle necessary for success as an athlete, and managing sport pressures while living within wider society. These latter subthemes are classified respectively as *discipline and sacrifice* and *the balancing act*.

**Table 2:** Themes and subthemes.

**Themes**	**Subthemes**
Conflating physical appearance and sporting ability	Social comparison in a sporting world
	Societal notions of ‘the athlete body’
Living as an athlete	Discipline and sacrifice
	The balancing act

### Conflating Physical Appearance and Sporting Ability

Understanding sport pressure in regard to physical appearance requires acceptance of the notion that body composition is inherently linked to sporting ability and, therefore, success. All the participants feel pressure from their sport to attain a particular aesthetic or body type for performance benefit. The following quotation from Callum, a triathlete, illustrates this: “*If you're lighter you tend to move quicker in the sport… The biggest benefit is to be as lean as you can really*.” The physical requirements for sport are seen by participants in terms of physics and practical requirements for success:

*If you did a mathematical equation, if you have two people who have the same acceleration but one is lighter, then the one who is lighter will win…people talk about the math and think if I lost five pounds then I could run that much faster per lap*. (Dan)

Participants feel they could see a performance benefit to being lighter and leaner for competition, but not without the need for strength. The following quote from Isabelle illustrates this idea: “*my sports all have required strength and speed … lacrosse particularly has so many other elements to it other than how light… you don't want to be that light as you get knocked over.”* Regardless of what the ideal body shape is, there is pressure to attain it.

This ideal body shape, dictated by sport pressures, means that participants often quickly conflate their appearance with what their bodies can do in performance. Henry discusses the expectation that comes with having the desired body shape for sporting success, saying, “*If you're light and you're thin, you're expected to be fast.”* This quote highlights that there is a disconnect between physical appearance and sporting ability, and that the two should not be seen as directly related. Nonetheless, Henry admits that the two concepts are often confused and that the perceived ideal clouds judgment:

*You often hear people say, oh, he looks quick or she looks quick. And I think you make that judgement based on how much body fat they're displaying and how muscular they look. I know for a fact that there's lots of guys there who look really lean and really fit and they look like they've just stepped off the Olympic podium, but I can beat them in a race, but then there's other guys who are bigger than me, heavier, they don't necessarily look like a particularly quick runner, yet they kick my ass in a lot of these races. So, I think our vision often gets clouded as to how we perceive a runner*.

The participants give voice to the pressure that sport creates to match one's physique with the one that science says will allow for maximum success on race day. For these athletes, the pressure to perform came with pressure to match the aesthetic most conventionally associated with success in their sport, with little room for the view that other body shapes may still allow for top sporting achievement.

#### Social Comparison in a Sporting World

Athletes are also susceptible to societal pressures that are often conveyed via an athlete's immediate and peripheral social sphere. These pressures heighten the conflation of physical appearance and sporting ability, as athletes compare their physique to others whose successes they are trying to emulate. As athletes are naturally competitive, comparing their own physical appearance to an athletic ideal and to other athletes is at the forefront during competitions. The interviews elicit the idea that comparison of physical appearance appeared to act as a stand-in for comparison of sporting ability. Participants describe using body type to measure up opponents and boost pre-performance confidence. Ben provides an example: “*When you go in for a race and you're standing behind the blocks, you look to other people to see what their build is as well and that influences how you feel*.” Furthermore, maintaining an elite level of athletic performance seemed to intensify this competitive “*culture of comparing between people”* (Isabelle), as there is an added pressure from others in sport to not only look like a stereotypical athlete, but an elite one at that:

*There's probably a bit of pressure in that once you've performed to a certain level, it's almost like you're expected to be at that level and getting better and that actually that level comes with an appearance or a body image and that's how everyone sees you*. (Georgia)

These comparisons to other athletes and an idolized ideal are magnified by the need for many sporting costumes to be figure hugging and aerodynamic; as Isabelle says, “*You spend a lot of time in a swimming costume, so everyone's checking each other out.”* The participants' experience indicates that the defined acceptable parameters for the physical appearance of an athlete became narrower as the competitive level increased, therefore also amplifying the confusion and conflation between physical appearance and sporting success. Furthermore, the costumes worn to enhance performance also enhanced scrutiny from other athletes and leave those performing vulnerable to the watchful eyes of society and their social sphere, which is where our analysis now turns.

The participants in this study discuss how further comparison, and the conflation between what the body looks like and what it can do, is worsened when it comes to viewing the accomplishments of role models. There is a sense that even athletes over-simplify sporting success in terms of the role that body shape, and by extension diet, plays in the achievements of those to which they aspire. The following two exerts highlight this idea with Georgia saying, “*These people obviously are the elites, are the best ones, so surely that's what we should all be like. So, it's almost like the role model type picture*.” This is further reinforced by Henry: “*There's this sense that they need to be thin and they need to be muscular and look like Jessica Ennis [Hill] or look like Mo Farah if they want to achieve that level of athleticism and success*.”

This culture of comparing body shape within the competitive world of sport is exacerbated by social media, particularly as the participant athletes spoke about populating online platforms primarily with accounts connected to sport. However, as with real life, athletes posted in costumes and poses that means what the body looked like quickly became a stand-in for what the body could do, and so emulating body shape is an understandable and perhaps inevitable next step. This idea is shown in quotes by two participants: “*On social media people do actually post quite a lot of pictures in their swimming costumes and all that stuff, and that is a factor in body image.”* (Francesca)

Georgia explains further:

*Like having almost like an idol that I want to be able to run like them and therefore I need to look like them, even without any text behind it, without any meaning behind the picture other than this is where I'm running, I think it automatically comes across*. (Georgia)

An intention to imitate physical appearance in order to imitate performance extended to copying training and dietary behaviors, as revealed by Alicia who says, “*Everyone else keeps theirs on the same platform, so I know what other people are doing and I have over the years a lot of people who I compare myself to.”* As well as by Dan who discusses the imitation in the following way: “*You are comparing yourself because you can see what other people are doing … everything is very visible, what people are doing and how they look or what they're eating.”*

From this it can be seen that to increase confidence in their own chances for sporting success, and despite professional athletic support, athletes turned to what they could measure, what they could see: physical appearance, much like lay people. Comparison of physical appearance to teammates, competitors, and role models is all exacerbated by social media and revealing costumes.

#### Societal Notions of “the Athlete Body”

Previous research has revealed that a lean ideal is prevalent among the general population; in addition, it has become apparent that this ideal gets applied specifically to how society believes athletes' bodies should look (Sundgot-Borgen and Torstveit, 2010). The societal notion of an athletic ideal exists despite a lack of knowledge regarding what body type is needed for success in sport and how this may vary from one sport to another. Participants describe how this societal ideal put pressure on athletes that is much greater than the pressure from other athletes, who understand the intricacies of body type and sporting success. According to Alicia, “*It's mostly from more kind of outside and I think the people who are the other athletes who train, [teammates] wouldn't be that judgmental*.” Emily agrees: “*I think the negative stuff does come more often from the non-athlete side of it for sure*.” Emily, as a rower, elaborates further:

*We're all very tall athletes, we're quite strong, have big shoulders, big legs, muscle and stuff like that, and often I think from the outside world or as a female particularly it's not the kind of social norm to be like a big strong woman really, but that's obviously what's required of your sport to perform at that level and so I think a lot of the time I do find myself thinking ‘oh this is not great, that I look like this’ because that's not how girls look even though that's what's made me great at my sport and stuff*.

The analysis shows that the compounded effect of sport and societal pressures to match this athletic ideal is inescapable. Even when the athletes are aware that looking slim and toned might not actually benefit their sporting performance, there remains societal pressure to fit an ideal. These expectations and ideals come from society's “*misperceptions and judgement and stereotypes* (Dan).” Henry adds that society seems to have a lack of understanding of what it takes to achieve sporting success: “*the general public misinterpret how much runners have to look after their diet and have to look after their general health and put training into achieve these times.”*

This pressure by society clouds the feeling of body positivity that stemmed from being proud that the body could achieve athletic feats, which Alicia describes here:

*I do feel I should look like a runner all the time. When I go to the gym, I should look in the mirror at the gym and have this ideal running shape which isn't necessarily being light, it's kind of like being strong and having this muscle tone…. sometimes I feel like I'm not lean enough, sometimes I feel I don't have enough muscle…. I have to remind myself it doesn't matter if you look a certain way, it's whether you can move quickly; that's the important thing and not if you look like you can run quickly*.

Emily echoes Alicia's sentiment, indicating that her past eating disorder derived from the pressure that society puts on athletes to match the image it has conjured: “*You think okay so I'm an athlete and there's a picture of an athlete; I need to look like that….When my eating disorder started …[it] probably came from society's image of an athlete.”*

The participants see social media as a place in which these social pressures are amplified, which allows for and even facilitates judgement of how they look, based on a faulty societal definition of an athlete. Alicia and Dan both emphasize this in their interviews:

*If you post on Instagram you feel like it has to have an image and then you feel a lot of pressure for it to be an ideal [image] and you spend an awful lot of time worrying about [it]*. (Alicia)

*I've met girls more than guys that will photoshop things and alter a photo of themselves or take something down straightaway if it gets a comment and I have seen some of the nasty comments people put on there…Particularly with social media, there's a lot of people now viewing people so there is judgment from all sides … comments like ‘this person doesn't look like an athlete’*. (Dan)

Henry highlights how photos in general, which are often showcased on social media, create pressure about body shape:

*In some races where I'd had my photograph taken, particularly ones where you're sweating a lot and your running vest sticks to your body, I'd sometimes see that little outline of that little bit of chub I've got around the belly and I felt I worried that if people saw that, they'd think god he's put weight on or how is he leading the race, he doesn't look like an athlete*. (Henry)

As seen from the extracts, these experiences demonstrates that living up to society's image of an athlete is intensified by the use of social media. The means by which social media operates also plays a role. Social media is a space defined by both anonymity and ease of communication, where almost anyone can connect directly to the personal devices in an athlete's pocket. For participants, this dynamic means that society's pressures are amplified.

*From social media you do get a lot of pressure because anyone can contact you and anyone can say whatever they want, so if they want to comment on how you look or anything like that, then they can*. (Emily)

Social media is a necessity for many of the athletes interviewed, as it influenced sponsorship opportunities; this is captured simply by Isabelle: *I'm not sponsored at the moment, I'm trying to get sponsored again, so that's why I would put stuff on social media*. Sponsorship, accessible mainly from social media, is key for allowing these athletes to continue training at their current level in a world in which many minor sports do not offer contracts and salaries that exceed living and training expenses. However, these sponsorships are the epitome of a conflation between what one looks like and what one can do athletically:

*I think there is big pressure on the way you look in sport and I know from before when it's been about getting sponsorship and things like that and you feel like the people who are awarded things are not always awarded on skill or that actually you are an inspiring athlete, it's usually quite aesthetic the way that opportunities get opened to you*. (Alicia)

Social media heightens societal pressures by inviting comments about the athletes' appearance based on what society equates with athletic success. These comments, which would otherwise have been left unheard by the athletes, are instead brought to the forefront of their daily scrolls through their social media feeds.

### Living as an Athlete

Analysis of the interviews highlights that to be a successful athlete, there are certain lifestyle—which includes food and behavior—that is expected within the sporting realm, the social sphere, and general society. There are certain behaviors that are simply a necessity when it comes to sporting success; specifically, discipline and sacrifice are required for sport participation. However, these behaviors may negatively affect social life, and are judged negatively by society when they fail to match that societal ideal. The narrative around discipline and sacrifice in sport is often extended to food choice, making diet and the function of food an inescapable part of the conflation between what the body can do and what it looks like.

For the athletes interviewed, food and its function as fuel, as well as the ability to manipulate food intake to access desired body shape, are connected to sporting success. This view stands in contrast to society's view of food as fun and its place at the center of many social events. Therefore, the discipline or sacrifice each athlete's sport required often means not partaking in social life, expected festivities, and what society dictated as normal, but still feeling a pressure to do so. The complex interaction between sport, the social sphere, and society manifested in the conflicting expectations around athletes' eating patterns. Society expects athletes to eat in a way that demonstrates their dedication to the athlete lifestyle, while still partaking in social events where food is the central focal point. The participants speak about internalizing these conflicting pressures, which are linked to disordered eating, and about needing to find a balance between them.

#### Discipline and Sacrifice

Recognizing food's function as fuel is an integral element of the athletes' disciplined lifestyle. All nine participants speak about the importance of eating enough food to satisfy training output and sport performance. For example:

*I very much see food-as-fuel and it's also definitely part of performing, it's like it's just another element, it's like doing strengthening and doing your aerobic training, it's just like that it's like doing your nutrition as well. So, I see it as a sort of component of my sport, it's not just something I do to survive*. (Emily)

*It's food for fuel…I'm just thinking how much energy is this going to give me, have I eaten enough for training…I cook things that will actually give me energy, not because it's tasty*. (Francesca)

Viewing food-as-fuel also means making a conscious effort to eat at the right times, fitting meals around training so that the fuel is utilized properly. Dan discusses the need for food to be “*digestible*” and well-timed so that “*when it comes to training, so not feeling sick when I'm actually in training and everything*.” This sentiment is echoed by Isabelle*: “when I'm training, I want to make sure that I've eaten enough and far enough in advance of training and that I've got food there to eat afterwards.”*

This careful focus on food to fuel training also means that food intake is manipulated to change body composition in line with an ideal that would help with sporting success.

In addition, because the athletes internalize the confusion about how they looked and how they performed, they sometimes prioritize eating to look a certain way over what might have been optimal for their performance or what their body is telling them.

*High level sport is more, it's like marginal gains isn't it; it maybe matters more what you eat…I think as an athlete I'm always thinking of how it's going to affect my performance*. (Francesca)

*There's been times where I get back from a long run and I think I just want to eat and eat and eat but then I think oh God, no I'm just adding weight and is my weight going to start going up, is that going to then affect my performance….I've got an odd relationship with food and I think it extends really from this sort of performance related side of things*. (Henry)

The participants describe how this manipulation of food intake has to be done with great care and concentration. Food has to serve its function as a crucial food source, but it also has to act as a body shape manipulator, both of which are perceived to aid training and performance. This is explained by Henry, when he says, “*there's almost this obsession with becoming as light as possible but not to the point where your body is no longer able to perform at its maximum ability*.” This notion is supported also by Georgia:

*There's a combination of thinking of food-as-fueling, and thinking about actually getting leaner effectively, so it was sort of working the balance out between those… I'm very aware that that body image, body shape has changed so my mind is going okay, well, I want to get back to how I was and therefore that leads to under fueling and then you're not getting the performance and the things that you need because you're trying to run on empty*. (Georgia).

To be successful, an athlete's lifestyle must involve prominent behaviors encapsulated as discipline and sacrifice. Discipline is considered a necessary and intrinsic part of elite sport. As Emily emphasizes: “*To get to this sort of level you do have to have a lot of discipline and a lot of … you have to be really strict with yourself*.” However, this discipline is linked to food and body image, and can be dangerous if taken to the extreme. For instance, when over-discipline is applied to diet, it can lead to disordered eating or even an eating disorder:

*I ended up being treated for an eating disorder because I had just decided that basically like food was a bad thing, and then having that super focused, super determined mindset, that's one of the reasons why I'm good at sport, but it's also one of the reasons why I was able to spiral quite quickly into an eating disorder*. (Emily)

The athletes interviewed consider the sport pressure of being disciplined and making sacrifices to be part of the game, but athletes also speak about how this often means missing out on fun or social occasions. Alicia says, “*You're spending your whole life trying to make things simple and make things so that you can train and recover but that also cuts out a lot of fun things*.” Francesca explains how this sacrifice and discipline extended to university life: “*I'm at university but I don't go out a lot … I've trained so hard to get to the fitness level I just don't want to ruin it.”*

As with the physical demands of sport, there is an inherent pressure on the athletes to lead a life that allows time for training and optimal fueling. These sport pressures demand a lifestyle that is disciplined and includes elements of sacrifice.

#### The Balancing Act

It is important here to reiterate the definition of society. In this study, society is defined as the non-athlete population, those who are aware of the existence of competitive sport and are perhaps also personally connected to it via friends, family, or fandom. Expectations from society are often juxtaposed with professional pressures about food-as-fuel to maximize training and performance. Athletes, therefore, have to negotiate these contrasting messages. The language used to describe this necessity is framed as “balance” by our participants; this balance is seen as an important part of being an athlete. The athletes acknowledge that the amount of food required for sporting success is far larger than for non-athletes, yet this need for extra energy sometimes drew unhelpful comments from those in the non-athlete realm:

*I think if someone was to come to a GB rowing camp with us and see the amount of food that we eat like five full meals a day, they would probably be quite surprised and probably would comment like ‘wow you guys eat so much food’. You could probably interpret that and think oh like maybe “I shouldn't eat so much food*.” (Emily)

Isabelle supports this notion:

*Society people often just think, why are you eating so much, and they just can't. That used to really bother me because I just randomly have like bowls of cereal throughout the day. And my parents, my dad would be like oh my goodness, what are you doing, you're going to get so fat*. (Isabelle)

Athletes in general are also asked to be fun and present members of a social sphere, which often involves eating meals with friends and family. Two opposing messages are internalized: the need to fuel the body for athletic endeavors vs. social pressures and expectations.

*I know what my goals are and what I need to do, so if I was in training really hard and I have some friends I want to go and hang out with and just blow off some steam and have a few drinks and it is someone's birthday and it seems fun and it is not going to be late and it is not like it's a massive deal, that would be fun… I think it's good to overall be a far more balanced individual, particularly for the long term; short term you can get away with it*. (Dan)

However, with food central to so many social engagements, the requirements for food to function-as-fuel and as a means to enhance performance is ever present:

*There's probably some conflict in that in one brain you're always thinking about, okay, well, what training session have I got tomorrow or when's my next race and how can I plan my social schedule around those and whether it be, you've got a group of friends that want to go out for dinner and actually you're thinking, well, I've got to do this session tomorrow and I know that going for X cuisine doesn't sit well with me and actually I want to do that session tomorrow and therefore the balance of do I/don't I?* (Georgia)

This balancing act, leads to internal conflict due to the tensions between a professional “normal” and social expectations. This appears to result in athletes' social spheres shrinking to include only others in sport (such as teammates and coaches). These social connections within the sporting sphere are then used to determine what diet would maximize performance. Participants describe the pressure that arose from their teammates modeling certain dietary behaviors:

*If your teammates are saying that they're doing this, they're eating this, or eating that, then you might feel pressure to do that as well*… [thinking that] ‘*if she's taking it, then I need to take it too to be as good as her*’ (Emily).

They also discuss the influence of coaches: “*Your coach has got these ambitions for you and he wants you to perform well, and does this relate to the way you look…and the way you view food*?” (Francesca). Pressure to copy teammates or follow a coach's suggestions regarding food and adjustment of body composition also exists.

The athletes speak about attempts to find an optimal, balanced behavior pattern that incorporate elements of rest, recovery, and fun, and include a more relaxed attitude toward food in order to be part of a wider social sphere. However, the balancing act implied between behaviors required for athletic success and those which compiled with social expectations is exacerbated because the athletes perceive that society has a double standard regarding discipline and sacrifice discussed in the previous subtheme. The disciplined schedule of an elite differs greatly from the norm for non-athletes and society at large, which sometimes lead to feelings of isolation. “*I think the thing I say most about being an athlete is it can be pretty isolating…I'm often here or there which means I can't really get a proper job*.” (Alicia)

Missing out on social occasions due to sport requirements is a required sacrifice. However, as Ben describes, society puts added pressure on athletes to maintain what it deems a “normal” social life: “*I couldn't go to many parties because I had a gala and they just didn't understand…they don't really understand the commitment [to] swimming.”*

Several participants report frustrations with society-as-a-whole because, despite often not understanding the intricacies or requirements of sport, society demands the impossible of athletes by asking that they make time for social, fun life elements, while also displaying discipline at all times. The participants explain that social pressures emanate from what society believes an athlete should do in terms of food and regime. Thus, when the athletes do exhibit a more balanced, relaxed approach in their food intake and behavior, several report that judgmental comments from society revert them back to the extreme. Emily and Georgia offer quotes that showcase this tension:

*People tell you that you're an athlete … so maybe you go out for a meal with your friends and then it's like often, like very often, people make a joke and they'll say, ‘hey you shouldn't be eating that you're an athlete remember’*. (Emily)

Georgia explains further, “*I think there's very much a little bit of almost like a stereotype that comes with it because you run or because you're active, then therefore you should be eating healthier food.”*

Athletes may have internalized this perceived judgment, which drives them away from balance and toward unhealthy levels of disciplined behavior that could be associated with disordered eating. Athletes may be forced, based on their sport schedules, to miss out on social events or even opt to avoid social situations to avoid this judgment. Participants describe how it is nearly impossible for them to find a balance between the requirements of their sport and the pressures non-athlete friends, or society at large, put upon them. This sometimes leads to feelings of trying to live up to an unmanageable double standard, and even feelings of negative affect and loneliness:

…*‘oh you shouldn't be doing this' or ‘why didn't you come out with us last night’. You need to manage that. And you can get that isolating loneliness or people don't understand why it's important to you too*. (Dan)

The athletes speak about how the required lifestyle and their intense interest in sport, combined with feeling misunderstood by society (especially for more obscure sports), means that they tend to shy away from talking about their sport or even engaging with non-athletes as part of their social sphere.

*They don't really understand the commitment swimming has to it, because as soon as you miss one session of swimming, you go back the day after and you just feel really different in the water … most of my time is spent with swimmers, I don't really spend much time away from them so my society is just swimmers in general*. (Ben)

This forced withdrawal from non-athlete society then puts added pressure on sporting achievement and success, as social life then depends on sport commitment. This sentiment is captured by Henry:

*If I'm no longer in that calorie deficit and my weight then starts to go up, if my body weight then starts to increase and I'm no longer running these quick times, I'm worried that am I not going to be able to train with that group anymore…When I decided to take running seriously I had an existing group of friends who I used to go out with at the weekend and we'd go out drinking, and we'd have fun, we'd do all these non-running related activities,[but] as I drifted more into the running community and I started to develop friendships there, I almost felt that I didn't want to be associated with the old me, the old life and that whole group of friends*.

In all the themes described above, comparison between the pressures from sport, the social sphere, and society created tension. This tension creates the conflation between what an athlete can do in terms of sporting success became inescapably linked to what they looked like, what they ate, and how they behaved. When discussing sport pressures alone, athletes speak about the physique required for sport empirically: a reality of sport. It is when they compared that physique to an ideal within sport—whether a role model or a past-self—or to teammates or social stereotypes, that disordered eating cognition and behaviors arose. This comparison is exacerbated by social media, which focusses on pictures and is used by athletes, their social sphere, and society more broadly, with little separation.

## Discussion

This qualitative work is part of a larger study aiming to examine Petrie and Greenleaf's (2007) model. The aim of the current exploration was to use thematic analysis to ascertain if athletes' experiences reflected key elements of the model, which posits that disordered eating is predicted in athletes by sport pressures and societal pressures. The thematic analysis was designed to explore these pressures in a range of sports and to better understand how they related to disordered eating, as these elements of the model were felt to be lacking in constitutive detail.

### From Societal to Social Pressures

Throughout the analysis, it became clear that so-called societal elements are so intertwined with the sporting world that they can be difficult to separate. This concept is best illustrated by Ben: “*My society is just swimmers in general*.” If an athlete's society is interwoven with their sporting world, differentiating between sport and societal pressures is ultimately difficult and problematic. With societal messages often delivered by those in an athlete's immediate social sphere, such as teammates, coaches, and role models, it can be challenging to establish whether the social elements of sport fit within or between sport and societal pressures (Cooper and Winter, 2017). This hints at the overlap between “sport pressures” and the social pressures that arise within sport.

Thus, what Petrie and Greenleaf (2007) term “societal pressures” are perhaps better framed as “social pressures” as understood and shared by the athletes themselves. Social pressures, in this context, are defined as those pressures that are experienced because sportspeople are located in their own social lives, first and foremost. In the original model, “societal pressures” denote concepts, specifically the thin ideal, that arguably enter the athlete's experience from “the outside world” and are then internalized by athletes and potentially influence disordered eating development.

The adoption of the term “social pressures” stems from the idea that pressures from society are not from a world outside of the athlete but are transmitted via social interaction with others. This can be seen unequivocally with the frequent reiteration of the word “people” by the participants. These “others” are ubiquitous and unspecified. The lack of precision is purposeful and important as it suggests these pressures may come from anywhere in the athletes' experiences. Our exploratory findings suggest that an athlete's social sphere, acts as a conduit for societal norms. Society espouses and shares widely held beliefs and norms, often ensuring conformity of all citizens through social pressures that communicate, validate and replicate prevalent, preferred values that permeate a person's social groups, irrespective of practice or profession (Levine et al., 2000; McNamara and Parsons, 2016). Embracing a more specific definition of social vs. societal allows for more nuanced interpretation of the findings presented here.

While the overall design and interview schedule were developed to allow deductive engagement with the two original pressures from the model, the research team embraced the spirit of qualitative analysis and allowed the analysis to elicit original themes. This revealed two themes, each with two subthemes that represent the pressures experienced by athletes. The themes and subthemes are: (1) conflating physical appearance and sporting ability, with subthemes of (1a) social comparison in a sporting world and (1b) societal notions of ‘the athlete body’; and (2) living as an athlete, with the corresponding subthemes of (2a) discipline and sacrifice and (2b) the balancing act. These findings are now discussed with reference to current understanding from literature with consideration of what this study can contribute.

### Conflating Appearance and Sport Performance: The Body Should Fit the Job

The first theme, conflating physical appearance and sporting ability, conveys the pressure to be successful in sport and the relationship between that success and the physical traits of an athlete. Body composition and body shape undeniably play a role in sporting success, and all participants were clear in their belief that there is an ideal body type for success in each sport. Participants espoused the belief that being lean but strong indicates a likelihood for improved performance, and they seek the “ideal” body type for sport via diet, despite knowing that a range of different body types may be associated with excellence in performance. Matching this ideal body shape is a pressure that increases with higher levels of competition. Furthermore, matching a physical representation of an athletic ideal is closely identified with sporting achievement which is amplified with the influence of social media, and with the pressure of getting and maintaining sponsorship. This finding at first seems to provide support for the inclusion of sport pressures in the theoretical model as a separate concept. However, exploration into the subthemes shows that it is the entanglement of sport pressures with social pressures that creates the most conflict with food, body image, and other concepts related to disordered eating. This idea is consistent with existing literature, where the notion that the pursuit of an athletic or performance ideal is linked to disordered eating, but upon critical examination, coaches, parents, and teammates have been found to exacerbate this link (Sundgot-Borgen, 1994; Thompson and Sherman, 1999a; Woods, 2004; Scott et al., 2019).

As shown in the first subtheme, social comparison of body types to teammates and sporting idols, as well as comments from coaches, increased the harmful confusion between what the body can do and what it looks like. This comparison of body types is intensified by revealing uniforms, consistent with findings of previous research (Thompson and Sherman, 1999a; Cooper and Winter, 2017; Greenspan et al., 2017). However, the participants were quick to add that the sporting community is more understanding than general (non-athlete) society, as athletes understood the need to be strong for their sport and the realities of how different sports lead to specific body types. As shown in the second subtheme, society projects a notion of a single, preconceived, slim-but-toned body type for athletes, despite no knowledge of which body type may be best for actual sporting success (Thompson and Sherman, 1999a). Pressure increases when participants thought about how they lived up to society's ideal athlete body, and comparison to someone who represented that ideal. The judgmental expectations from wider society of how an athlete should look are not only harsher and more hypercritical than those within the sporting world, but they were also amplified by social media. Previous research, like the current study, has shown that the internalization of and drive to match stereotypical social expectations is associated with disordered eating in athletes (Anshel, 2004; Cooper and Winter, 2017).

Understanding the role of social interactions within sport, as well as the inescapable social commentary on athletes' bodies, shows that sport pressures do not exist in isolation. In fact, it is this entanglement of sport and social pressures that appears to be a driving force behind disordered eating development. Previous qualitative work also highlights how the interaction between sport and social pressures—for example, teammates modeling disordered eating behavior at communal meals, or comments from parents and coaches encouraging loss of body fat for better athletic performance—is related to disordered eating development (Woods, 2004; Papathomas and Lavallee, 2010; Arthur-Cameselle and Quatromoni, 2011; Cooper and Winter, 2017; Scott et al., 2019). Other qualitative research that has found that the elements contributing to disordered eating symptoms in an athlete's life are not necessarily unique to athletes (Arthur-Cameselle and Quatromoni, 2011, 2014). Quantitative work has exposed elements of the social sphere common to non-athletes and athletes alike, such as attending an elite school and experiencing social pressure, revealing that some of the disordered eating risks posed to athletes may not be sport-specific (Krentz and Warschburger, 2011; Pettersen et al., 2016).

Additional pressure on athletes stems from social media, and specifically from the reality that sponsorship deals are often based on having a body type that fits society's athletic ideal. Thus, athletes feel both psychological and financial pressure to attain that body type, rather than what might be optimal for them as a performing athlete. This study was based in the UK, where sponsorship deals are often career-determining for athletes; this contrasts with research based on student athletes within the NCAA university system in the USA, who do not rely on sponsorships, and where much of the previous research into disordered eating in sport has been located. The inclusion of social media in this investigation was relatively novel in this specific area of research, and was an important innovation in view of existing research which shows that social media is used for online interactions between athletes and non-athletes, including comments and chats (Pegoraro, 2010). Originally a unique theme of its own, social media was instead determined to be a constant concept interwoven throughout the themes and subthemes, intensifying the interaction between sport and society. Asking about the use of social media revealed that athletes primarily fill their online feeds with communications from other athletes. However, social media does not exist solely in the sporting world, and the need to adhere to society's expectations online led the athletes to feel as though they needed to portray, or even change, their bodies in accordance with society's image of an ideal athletic body. As the use of social media continues to escalate, including measures of social media usage in future research is clearly warranted.

### The Sacrifice and Balancing Act of Living an Athlete's Life

The second overarching theme, the experience of living as an athlete, is something that every sport participant takes forward into their daily social interactions. An athlete's lifestyle is essential to success, and the discipline and sacrifice inherent to that lifestyle are carried over into daily activities as well as food choice. The day-to-day diet of an athlete requires substantial attention to the quantity, timing, and nutritional value of each meal. This finding corresponds with previous work that also highlights the idea of food-as-fuel, with the athletes in this study sharing similar experiences of creating a diet that would allow them to train and compete at their best (Lunde and Gattario, 2017). Viewing food as a performance aid and as something that can help manipulate body shape is intensified by the conflation of what the body looks like and how it performs. Additionally, a disciplined lifestyle featuring sacrifice for the sake of sporting success is widely considered an integral part of sport, and has been tied to behavioral tendencies related to eating disorders and disordered eating (Thompson and Sherman, 1999b; Nesti, 2007).

The next subtheme, “discipline and sacrifice,” encapsulates daily choices made by athletes. Despite the regime that undergirds life as an athlete, there is still an expectation that athletes should participate in the food-centered activities and structures that society deems normal (Beardsworth and Keil, 2002; Jastran et al., 2009). Whether this “normal” is the routine of eating three meals per day or going out for a celebratory dinner, athletes feel they are expected to fit these into aspects of daily life even if they are not optimal for athletic endeavors. This tension compelled the athletes to attempt to find a delicate balance between eating to improve their sporting performance and matching social expectations.

As discussed in the final subtheme, athletes seek to create a balance between the extremes required for elite sport and normal daily life. The athletes often found that a typical social life is unobtainable and felt that whenever they did exhibit a more relaxed approach to diet and other behaviors, they are pressured by society to return to extreme displays of discipline to better fit the societal definition of an athlete. Previous research has found that athletes make more extreme and unhealthy sacrifices to match social ideals or satisfy social elements within their sporting world (Hughes and Coakley, 1991; Waldron and Krane, 2005). Additionally, social isolation as a consequence of elite athlete lifestyle, and an inability to find balance, was observed in this study and is consistent with previous research in sport (Pinkerton et al., 1989). However, the athletes' attempts to moderate extreme behaviors required by sport to create more balance in their lives, only to be driven back toward extremes by social ideals, has not been reported before in the literature. Living as an athlete and the related first-hand phenomena that are captured in that experience are not part of the T1 model as separate factor, but instead make up the landscape in which disordered eating behavior and cognitions develop (Stoyel et al., 2020).

The conclusion that it is the interaction between sport pressures and social pressures that is most likely to increase the risk of disordered eating symptoms and related factors does not suggest that engaging in sport has no impact on eating behavior. Rather, these athletes experiences indicate that sport pressures alone are not perceived as the decisive element. Society's inescapable messages of what an athlete should attain in terms of body shape, diet, and lifestyle, which are often inculcated by the social sphere within and without sport, appear to be what leads to disordered eating issues. Sport is the context in which social pressures take place. Therefore, it could even be posited that, in a revised model (Figure 2), the factor labeled “societal pressures” actually represents a tangled interaction between sport and social pressures, with no athlete immune to social pressures that pervade the rest of their lives, including their profession.

## Implications and Future Research

These findings offer some tentative clues as to why sport pressures no longer fit the theoretical model when applied to a large and diverse sample of athletes in the quantitative phase of this project (Stoyel et al., 2020). Due to the qualitative nature of the current study, the aim of this study was never to confirm or disconfirm the entire model. Future research should explore the role of other elements of the model, such as body dissatisfaction and internalization of messages from others and ideals, qualitatively, examining their connection with disordered eating symptomatology. Given that social pressures appear to compound the effect of sport pressures on athletes, interventions should be aimed at coping with these social pressures, especially when social media allows for total omnipresence. This finding potentially means that interventions and treatment options for disordered eating devised for the general population could be adapted to work with athletes, as long as they incorporate an understanding of the specific pressures on athletes and how they may fit with or contradict social pressures. For example, modifying social media usage or education about resisting unhelpful social messages, both in person and online, may a good place to start. As athletes are already using online platforms, an intervention that exists on these platforms could have widespread reach and impact (Guest et al., 2019). When working with athletes, it important to realize that the pressures they feel are not isolated to sport, as has been shown in previous work; instead, disordered eating thoughts and behaviors appear to arise from a complex interaction of risk factors and correlates that can exist both in and out of sport (Striegel-Moore and Bulik, 2007).

It is also important to address that despite previous histories of eating disorders and disordered eating, participant EDE-Q scores were not at clinical levels. It is possible that this is because the questions asked did not take into account the realities of being an elite athlete. This discrepancy is highlighted in this study: lead author of this research and conductor of the interviews is a practicing sport psychologist, which meant that sufficient rapport was built such that one male participant disclosed in the interview for the first time that he believed he had an eating disorder, yet his EDE-Q score does not reflect this self-diagnosis. Elite sport requires extremes, such as over 15 h a week of exercise, or micromanaging food intake for the sake of performance. These “extremes” that are normal to sport may present as pathologic in clinical contexts. Therefore, scales and diagnostic tools must take into account sporting endeavors. For instance, an athlete may score low on an EDE-Q question about going long periods without eating, due to the fuel requirements of their sport, but it is possible that that score may mask underlying pathological cognitions.

### Limitations

While this study and overall project is strengthened by the inclusion of male and female participants, this study was limited by the lack of diversity in the sports represented, with the vast majority categorized as endurance racing sports. This is noteworthy, as the training load of these sports influences body shape such that female participants might, in fact, more closely match ideal societal standards, compared with those participating in more strength-based sports, such as weightlifting (Byrne and McLean, 2001). Additionally, it is important to recognize that our number of interviewees fall short of Braun and Clarke's own recommendations for optimal “sample size” of 10, however the research team feels confident that data saturation was reached (Braun and Clarke, 2013). Furthermore, most of the interviews were conducted virtually, and while previous research has deemed this an acceptable practice, the ability to build rapport within a single interview, especially a virtual one, might have affected the openness of the responses (Janghorban et al., 2014). This is particularly true of openness or disclosure on a subject that is sensitive, and many athletes worry about disclosure of any disordered eating due to stigma (Papathomas and Lavallee, 2010). Finally, due to the timing of this project, the final three interviews took place at the start of the Covid-19 pandemic, which might have led to unusual eating or exercise practices. To check for this, participants were asked whether their experience during the pandemic lockdown influenced their view of food as an athlete; however, they did not report anything of consequence for this study.

### Conclusion

Overall, the findings of this study indicate that there is a tendency to assume that looks and lifestyle are synonymous with sporting success, and that a successful athlete is one that looks like society's image of such, behaves like one socially, and eats like one. It may be that living up to this concept of a successful athlete, combined with the scrutiny from the social sphere, is linked to the development of disordered eating behavior in athletes. Participants describe how the conflict between sport and social pressures increased the risk of disordered eating, rather than discrete sport pressures alone. The unhelpful fusion of physical appearance and sporting success has sent heightened messages from society to these athletes. Striving to match a potentially unhealthy aesthetic to attain success in sporting competition is not a winning combination.

## Data Availability Statement

The datasets presented in this article are not readily available because: The data set is qualitative and as such may have identifiable information. It is pseudonymised rather than anonymised. Should the data be requested, edited versions of this data can be shared. Requests to access the datasets should be directed to hannah.stoyel.13@ucl.ac.uk.

## Ethics Statement

The studies involving human participants were reviewed and approved by UCL Research Ethics Committee Approval ID Number: CEHP2018573. The patients/participants provided their written informed consent to participate in this study.

## Author Contributions

HS is author and creator of this project and manuscript. RD assisted in analysis and many re-drafts and edits of manuscript. AS assisted in analysis and many re-drafts and edits of manuscript. VS-F and LS acted as formal supervisors and both helped with writing and revisions of manuscript. All authors contributed to the article and approved the submitted version.

## Conflict of Interest

The authors declare that the research was conducted in the absence of any commercial or financial relationships that could be construed as a potential conflict of interest.
